# New Approaches in Urban Forestry to Minimize Invasive Species Impacts: The Case of Xiongan New Area in China

**DOI:** 10.3390/insects11050300

**Published:** 2020-05-12

**Authors:** Hui-Ping Li, Jacob D. Wickham, Kathryn Bushley, Zhi-Gang Wang, Bin Zhang, Jiang-Hua Sun

**Affiliations:** 1 Key Laboratory of Forest Germplasm Resources and Forest Protection of Hebei Province, Forestry College of Hebei Agricultural University, Baoding 071000, China; huipinglipaper@163.com (H.-P.L.); wzhg1956@163.com (Z.-G.W.); 2 State Key Laboratory of Integrated Management of Pest Insects and Rodents, Institute of Zoology, Chinese Academy of Sciences, Beijing 100101, China; jacobwickham@ioz.ac.cn (J.D.W.); sunjh@ioz.ac.cn (J.-H.S.); 3 Department of Plant Biology, University of Minnesota, Saint Paul, MN 55108, USA; kbushley@umn.edu; 4 CAS Center for Excellence in Biotic Interactions, University of Chinese Academy of Sciences, Beijing 10049, China

**Keywords:** Xiongan New Area, urban forest, pest invasion, pest management

## Abstract

China is implementing an extensive urban forestry plan in Xiongan New Area (XNA), a new city in Hebei province. The city has been designated to serve Beijing’s noncapital functions and promote the integration of the broader Beijing–Tianjin–Hebei city-region. As part of a green initiative to minimize environmental impacts and its carbon footprint, a massive urban forestry system has been planned on an unprecedented scale, expected to cover over 600 km^2^ by 2030. Using science to inform policy, one major goal is to simultaneously minimize impacts of invasive species, while making urban forests more resilient to potential invasive species threats. In this review, we introduce these urban forestry plans such as basic concepts and principles for afforestation, tree species to be planted, delineation of existing pests already established, and expected forest invasive species of concern threatening the new area. Finally, we introduce a framework for invasive pest management strategies in XNA based on a “big data” approach and decision system to minimize impacts of invasive species. This new approach to urban forestry has the potential to become an exemplary global model for urban forestry planning, one that integrates research activities focused on forest health surveys and monitoring with sustainable forestry management. Finally, we provide an overview of the forest health policy required for the design of an unprecedentedly large new urban forest from initial planning to full implementation of an integrated forest management program.

## 1. Introduction

Urban forests are beneficial to human health and well-being as well as preserving urban environments and biodiversity [1,2,3]. Despite these benefits, urban forests, especially those in close proximity to international ports, often harbor abundant invasive pest species [4]. Several factors can contribute to urban forests becoming hotspots of pest invasion [5]. Initially, the high density of people and proximity to transport linkages (e.g., airports and seaports) enable the arrival of new species through dispersal pathways such as trade, tourism, and horticulture [6]. Consequently, urban areas are under high risk and may experience high propagule pressure, meaning they experience high frequencies of introductions of non-native species [7]. Moreover, globalization of economies and climate change also significantly contribute to the increase of exotic urban forest pests [8]. In addition, the urban forest itself is a key facilitator for pest invasion due to presence of suitable habitats for invasive pests, such as diversity and often non-native host tree species, heterogeneous landscapes, and favorable microclimatic conditions (e.g., urban heat-island effects) that could enhance the opportunities for the establishment of non-native pests [9,10].

Invasive pests cause a variety of detrimental impacts to urban environments and human well-being. For example, invasive pests could directly cause substantial economic losses by devastating valuable ornamental trees. Massive losses of urban trees may indirectly result in destabilizing entire urban ecosystems, reducing residential property values, and can even negatively affect human health [11,12]. Furthermore, once established, populations of invasive pests can be amplified and the urban forests may serve as “bridgeheads” to threaten natural forest landscapes far beyond the urban forest [13]. Inevitably, such invasions lead to costly and challenging eradication programs and post-establishment management strategies [14,15]. In view of this, policies and strategies have been extensively discussed in many large or historical cities around the world to manage invasive pests [12,16,17,18]. However, thus far, there has yet to be any policy or strategy implemented for a new city with a freshly planned urban forest.

In this paper, we explore both the challenges and opportunities for novel approaches for invasive pest management in a new city by examining the unique case of Xiongan New Area, China. We also unveil an urban forestry plan with respect to this special zone (e.g., what tree species are being planted, existing tree species, and expected forest invasive pests) and introduce the strategies to manage potential invasive pests. Our main objective is to utilize the Xiongan New Area as a case study to address how planners and resource managers can create an extensive urban forestry system with the goal of both minimizing impacts of invasive species and making urban forests more resilient to potential invasives, and to provide an overview and critique of the Forest Health policy.

## 2. Xiongan New Area

**Figure 1:**
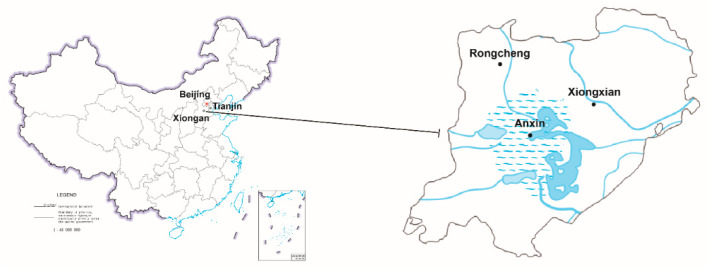
District map of Xiongan New Area in China. Xiongan New Area is located in the central region of “Jing-Jin-Ji” region, situated 120 km from Beijing and 110 km from Tianjin. It mainly includes Xiongxian, Anxin County and Rongcheng County with an area of 2000 km^2^.

On 1 April 2017, China launched a “Millennium plan” to create a new city of Xiongan New Area (XNA), which includes Xiongxian, Anxin County, and Rongcheng County of Baoding, Hebei Province, located roughly 120 km southwest of Beijing. It will initially cover around 100 km^2^, expand to 200 km^2^ in the middle stage, then eventually cover 2000 km^2^ with a population of between 2 and 2.5 million (Figure 1). According to the master plan published on 21 April 2018, XNA will be the next new area of “national significance” following the Shenzhen Special Economic Zone and Shanghai Pudong New Area in China, and will perform the non-capital functions of the capital city Beijing, such as headquarters for global companies and enterprises. The endeavor explores a new model of optimized development in densely populated areas and promotes the integration of the Beijing–Tianjin–Hebei city-region [19]. In light of the “big city malaise” (e.g., traffic and environmental problems) typical of previous urbanization, policymakers have made ecological and green development the top priority in the creation of XNA [20].

## 3. Millennium Show Forest

**Figure 2:**
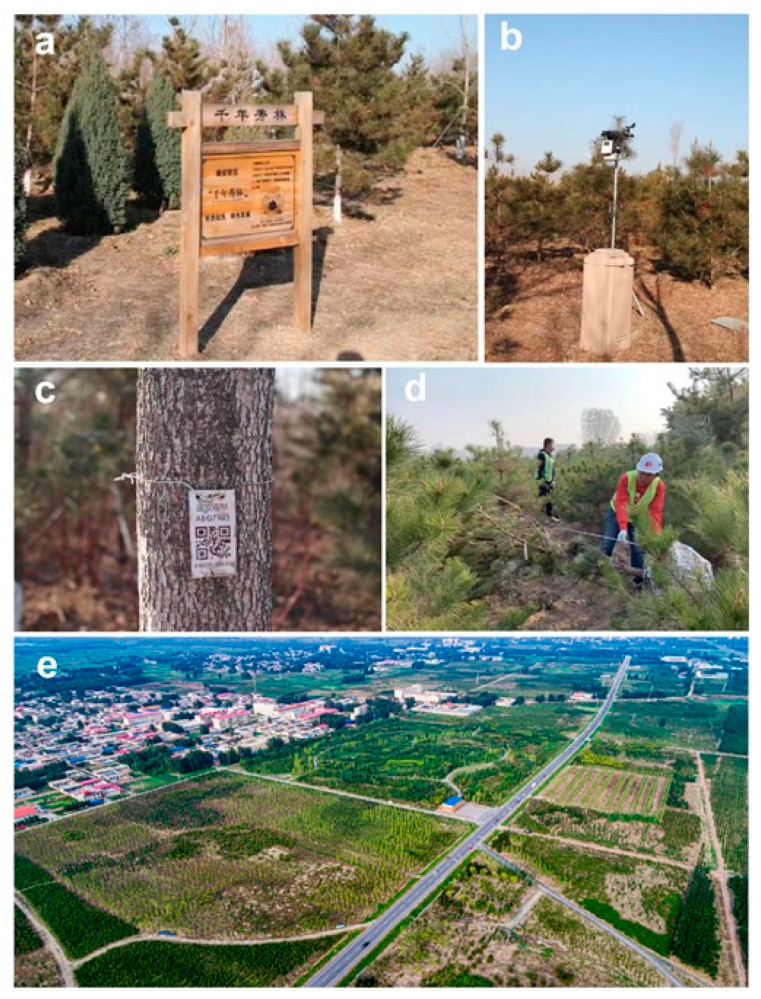
Exemplary landscapes, techniques, and criteria exhibited during afforestation in Xiongan New area. (**a**) The introduction of “Millennium show forest” with special emphasis on prioritizing sustainable ecological and green development. (**b**) Miniature meteorological monitoring and intelligent irrigation systems used in the “Millennium show forest”. (**c**) Two-dimensional QR code, the ID card for each tree for tracking its information on species, life cycle, sources, planting time, and style, including the maintenance record from nursery to planting and management. (**d**) Measuring the height before planting a pine tree. (**e**) Aerial view of a section of planted urban forest in Xiongan New Area.

As part of prioritization of ecological and green initiatives, a massive urban forestry system, termed the “Millennium show forest”, has been planned. Unlike a general urban afforestation project, this project follows the principals of natural forest succession to construct a close-to-natural urban forest composed of mixed-aged, multi-layered (canopy, mid-level, and understory), mixed species forests planted on a “virgin” land, with a goal of reaching a final coverage of over 600 km^2^, representing 30 percent forest coverage in the total planned 2000 km^2^ XNA area by 2030. The “Millennium show forest” will develop together with the XNA to form various landscape patterns for scientific research and ecological landscape preservation. This urban forest will also provide an important ecological buffer zone and will promote ecosystem services such as supporting maintenance of natural water and nutrient cycles, and improve human well-being with both spiritual and recreational benefits as a shared space for future citizens (Figure 2a). 

Since the program commenced on 13 November 2017, great progress has been achieved as a total 12 million seedlings have been planted over a 113 km^2^ area thus far. Afforestation was carried out with careful attention to plant tree species that were compatible with particular sites (e.g., soils, slopes, elevation). Moreover, to guarantee a high quality of urban forest, the local government compiled the “Manual of afforestation work in XNA”, which sets high and strict standards for project organization and command, land use, bidding from forestry contractors, species and seedling selection, excavation, packaging, unloading, planting, irrigation, and subsequent management and protection (Figure 2b–d).

The following are exemplary criteria or new techniques to be implemented in the afforestation program: (1) the tree species must match with the land variables so that chosen species are adapted to the natural abiotic conditions such as soil pH, water content, and precipitation specific to North China, therefore the use of local species and seedlings are prioritized; (2) a more natural scattered planting technique is employed according to strict protocols on quantity, density, and height (Figure 2d) to simulate more natural distributions of trees rather than a traditional patterns of planting in rows and columns; (3) implementation of automatic management techniques, including miniature meteorological monitoring and intelligent irrigation systems, are widely applied in this forest (Figure 2b); (4) collection of “big data” to form a network where each tree has its own “ID card” (two-dimensional QR code), which could facilitate long term monitoring to track metadata such as species, life cycle, sources, planting time and style, origin, and maintenance record from the nursery to transplanting, and finally management (Figure 2c). Data collected include tree ID (genus ID, species ID, tag number, location, height, diameter), planting details (nursery origin, planter, date of planting), maintenance record (weeding, watering, pest control treatment, date of maintenance, maintenance worker), and preferred environmental site conditions for that species (humidity, soil, elevation, etc.). Based on these fundamental pieces of information, a “digital forest” will be constructed by developing a Xiongan forest “big data” system to integrate all these data using cloud-computing in real time. The trackable digital forest will provide valuable knowledge such as natural forest succession and a real-time survey and monitoring program to detect and track invasive species establishment and impacts to guide the pest management program. In addition, permanent plots will be established to provide long term data on tree growth, invasive species monitoring and forest health.

To form this multiple layered forest of canopy, mid-story, and understory trees, 174 species of plants belonging to 37 families and 86 genera ranging from shrubs to deciduous and evergreen trees were used for this planted forest (Figure 2e; Table 1). A total of 111 species of deciduous trees and 14 species of evergreen trees form the overstory (or upper layers), and 39 species of shrubs contribute to the understory (or lower layers), while 10 tree and shrub species with intermediate height comprising the middle-layers (Table 1). Some of the trees are North American and European in origin and have been widely planted as ornamentals in China for decades; however, the forest managers must be cautious and extra effort taken to monitor them for attack from both invasive and native species, much like the sentinel trees planted in other cities in East Asia [21]. 

## 4. The Threat of Invasive Pests in XNA

Given large-scale and rapid development of afforestation of this new city, invasive forest pests are expected to be a serious problem facing the “Millennium show forest”. On one hand, as a result of the movement of a large number of seedlings, building materials, and people into XNA, the city and its surrounding forest will be at high risk of introduction of potentially harmful invasive species. On the other hand, the pests that are already present in XNA are also an important source of invasive species in the new urban forest lands. 

### 4.1. Existing Forest Pests

According to our preliminary investigation, there are 37 species of forest pests that include 18 diseases and 19 species of insects and mites already present in XNA (Table 2). Among insects and mites, the longhorned beetle, *Semanotus bifasciatus* (Cerambycidae), causes the most serious damage to trunks and branches of *Sabina chinensis,* with a 35.4% infestation rate. The snout moth, *Dioryctria rubella* (20.7% of infestation ratio) severely harms buds of *Pinus tabuliformis*. In addition, the infestation rates of *Thysanogyna limbata*, *Hyphantria cunea*, and *Panonychus ulmi* that forage on various broad-leaved trees are all more than 20%, respectively (Table 2). The prevalence of leaf diseases, including needle cast of *Pinus tabuliformis*, leaf blight on *Ginkgo biloba*, leaf spot of *Paeonia suffruticosa* and *Paeonia lactiflora*, and iron deficiency chlorosis of *Malus* trees, have a 15.8%, 15.2%, 16.5%, and 15.9% infection rate, respectively. Moreover, some diseases can also damage trunks and branches (rot, ulcer and gummosis diseases of broad-leaved trees), roots (cherry root carcinomatosis), and even the whole plant (blight of *Cotinus coggygria*, *Acer truncatum*, and *Gleditsia sinensis*) (Table 2).

### 4.2. Potential Invasive Forest Pests

In addition to the local pest species, other important invasive forest pests causing devastating damage to forests in China and worldwide have the potential to threaten the XNA as sources for prospective invasion and outbreak. These include pinewood nematode (PWN) and red turpentine beetle (RTB).

Pinewood nematode, *Bursaphelenchus xylophilus* (Steiner and Buhrer) Nickle, the causative agent of pine wilt disease (PWD) and a global quarantine pest, is native to North America, but was initially introduced to Japan in the early 1900s, and subsequently to China, Korea, and then to Europe, including Portugal and Spain [22], where it causes devastating damage to conifer forests, particularly *Pinus* spp. For successful invasion, PWN also engages in a symbiotic partnership with *Monochamus* spp., its beetle vector, as well as associated bacteria and ophiostomatoid fungi [23].

In China, the nematode was discovered for the first time in Nanjing in 1982, then rapidly spread into 588 counties in 18 provinces in the next three decades, and has killed more than 9.74 million ha of pine forests. With losses of more than US $20 billion, the PWN has become the most destructive pathogen of forests in China [23,24]. So far, XNA, located in Hebei province, is still PWD-free, but is considered high risk for potential PWN invasion for the following reasons: First, XNA is surrounded by epidemic areas including Shandong, Henan, Tianjin, and Liaoning provinces. Second, many pine trees including *Pinus bungeana*, *P. strobus*, *P. thunbergii*, *P. armandii*, *P. ponderosa*, *P. tabuliformis*, and *P. sylvestris* represent the most important tree species in the urban forest ecosystems in XNA, and most are primary hosts for PWN. Finally, along with the development and natural succession of this urban forest, *Monochamus alternatus*, the symbiotic partner and main vector of PWN in China, will inevitably be introduced into XNA as predicted by previous studies [25].

The red turpentine beetle (RTB; *Dendroctonus valens* LeConte) is a secondary pest of pines in its native range in North America (Canada, the United States, and Mexico) and parts of Central America (Guatemala and Honduras), where outbreaks and tree mortality attributed to RTB alone are rare. In the early 1980s, RTB was first detected in Shanxi Province, northern China, and was accidentally introduced on unprocessed logs imported from the west coast of the United States. Since 1999, RTB has spread rapidly to adjacent provinces, including Hebei, Henan, Shaanxi, and Beijing, and most recently to Tianjin, Liaoning, and Inner Mongolia [24], causing unprecedented damage by killing more than 10 million healthy Chinese pines (*P. tabuliformis*) as well as other pine species such as *P. bungeana* [26,27]. The complexity of interactions that range from antagonism to mutualism among RTB, its symbiotic fungi *Leptographium procerum*, gut-associated bacteria, and host plant trees greatly contribute to RTB’s invasiveness and destructiveness [28]. In addition, human-mediated dispersal also plays an important role in RTB’s invasion [24].

Currently, the XNA is RTB-free, mainly due to the absence of clustered pine trees in the area. However, along with the planned large-scale development of afforestation, especially *P. tabuliformis*, the area will be easily attacked by RTB as it is a prevalent species in pine forests of Hebei province near XNA. At the same time, increased populations and extensive exchange of goods and people during urbanization will further increase the risk of RTB invasion.

In addition to these two major potential invasive species, the Asian longhorned beetle (*Anoplophora glabripennis*) and Emerald Ash Borer (*Agrilus planipennis*) are expected to become invasive pests of concern. These are considered native species; however, due to planting of both native and non-native trees, these are expected to become a problem as the forest becomes established. There is already noticeable evidence of *A. plannipenis* attacking Chinese white ash (*Fraxinus chinensis*) throughout the Olympic Forest Park in Beijing (with size of 6.8 km^2^), widely planted in the park during the 2001–2008 development running up to the 2008 Olympic Games, and there were also plantings of European and American species of ash trees in the vicinity of Beijing, thereby acting as sentinel trees (Personal observation by J.D. Wickham).

## 5. Strategies of Invasive Pest Management

Managing invasive pests in urban forest is often more complicated compared to traditional pests in agricultural or natural ecosystems because of the diversity and complexity of social environments such as divergent opinions of stakeholders on particular species [17]. Unlike standard urban forests, the “Millennium show forest” in XNA, however, possesses some advantages for invasive pest management. This project was thoroughly controlled by the local government from its initial planning, afforestation, to management, thus making it much simpler to incorporate decision-making protocols with regards to invasive pest management by minimizing the challenges posed by socio-political factors. In addition, collection of “big data” such as tracking records of each tree will provide valuable information for risk evaluation and greatly assist the managers in designing appropriate strategies when actions need to be implemented.

**Figure 3:**
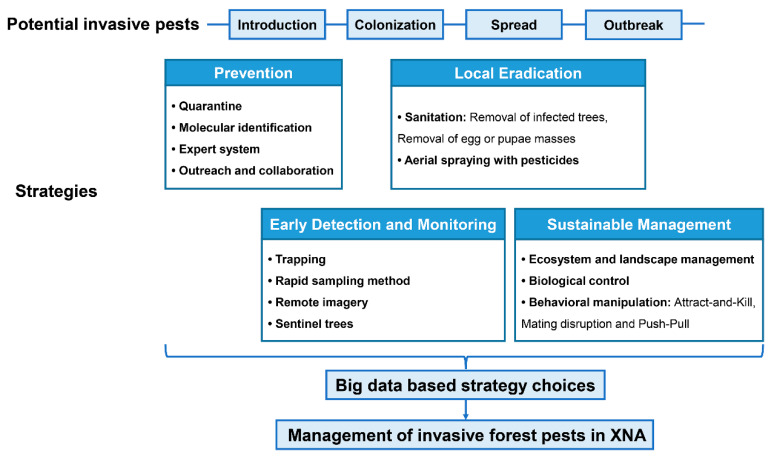
The framework of invasive pest management in Xiongan New Area (XNA). Four main strategies including prevention, early detection and monitoring, local eradication, and long-term sustainable containment implemented for different stages of the process of pest invasions. We also listed specific techniques for each of the strategies that can be selected based on a “big data”-based decision system to minimize the impacts of invasive pests in Xiongan New Area.

Based on these features of the “Millennium show forest”, we suggest a framework for management of invasive forest pests in XNA to minimize the impacts of invasive species on this urban forest (Figure 3). Within this framework, four complementary strategies, including (1) prevention of new introductions, (2) early detection and monitoring of invaders that cannot be prevented, (3) local eradication of isolated populations, and (4) long-term containment of well-established invasive species, are essential. These strategies form multiple lines of defense to block the progress of biological invasions (i.e., introduction, colonization, spread, and outbreak). From the ability to collect an unprecedented amount of real-time data, this “big data” based decision making system for making strategic choices in response to invasive species will be a major component of this framework.

Each tree has a QR code containing data on the tree’s provenance, current health, and invasive pest status. In order for the “big-data based” strategy to be useful and successfully meet the long-term forest management goals, the individual tree data will be made publicly available and integrated with risk maps for each potential pest species showing (1) host suitability, (2) host location, (3) current pest range and (4) detections, and (5) current management actions (quarantines, sanitation, biocontrol, pesticide spraying). To engage the public, each area should have a list of potential invasive species at information kiosks or nature interpretation centers, or integrated with smart phone applications, enabling potential citizen scientists to make contributions such as reports or sightings of invasive species. Currently the live, interactive database is accessed via an Android App under the name Xiongan Forest APP (searchable “Xiongan Senlin” in pinyin). Together with a planned research center led by Hebei Agricultural University and in cooperation with the local Forest Pest Station, the existing data and risk maps will be curated and integrated with new data from permanent plots in real-time, thus enabling forest protection managers to make science-based recommendations and management actions for invasive species management. In the future, this unique framework of the management system, which utilizes an integrated “big data” approach and decision system, will continually generate data on forest health and succession, and provide a real-time database for prevention, monitoring, and management of pest invasion.

### 5.1. Prevention of New Introductions

Introduction is the first stage of invasion, and preventing the initial arrival of an invasive species is a preferred and effective strategy. As forest pests and diseases are usually introduced unintentionally [29], a system of strict quarantine procedures should be implemented at port facilities or at the main intersections during the trade of nursery plants, goods, and construction materials into XNA.

A pre-requisite to any control measures on invasive species is the ability to rapidly and accurately identify the potential invasive species. Besides the traditional techniques for species identification, new and easier approaches using molecular biology, biotechnology, and digital imaging are also encouraged for early detection and identification. One such powerful tool under development is DNA metabarcoding via high-throughput sequencing [30]. DNA metabarcoding can provide millions of sequences from a bulk sample and is sensitive to detection of “rare” insect species in a sample [31,32], such as detection of a single individual invasive species, which makes it a promising approach to increase speed and lower the cost of detecting a new invader. Combining genetic information with improved digital imaging could provide powerful tools for future diagnostic identifications.

To meet the pressing need for rapid, flexible, and scientific screening or imported materials, expert systems that automate risk identification in ports also need to be developed. Such systems have been widely applied in US forests for reducing the threat of invasive species [33]. By integrating data on product records, receiving port, destinations, and habitat distribution (e.g., climate, soils, forest types) in XNA and pest distributions in originating regions, this system could automatically target inspections on certain commodities or other potential vectors known to harbor high-risk species or from materials that come from areas with invasive species outbreaks.

### 5.2. Early Detection and Monitoring

Early detection is crucial for successful eradication. Active surveillance and monitoring for invasive forests pests should be focused on trees in urban and suburban areas. Botanical gardens, arboreta, and woodlots that harbor high plant diversity, and those that are close to transport linkages should be given particular priority in structured surveys and trapping networks [5]. Trapping surveys using multicomponent lures such as sex or aggregation pheromones is a green technology, species-specific, and effective method for early detection. This method has been widely used in early detection of invasive forest Lepidoptera and Coleoptera [34,35,36].

The innovation of rapid sampling methods for potential invasive forest pests will also be developed within this framework. For example, we have developed a novel rapid sampling method for pinewood nematode (PWN), *B. xylophilus*, using the chemotactic response of PWN to its insect vector, *Monochamus alternatus*. This method can effectively detect PWN from infested trees within a 2 h trapping period [37], and thus can promote both early detection and long-term monitoring.

Remote sensing can also aid in monitoring certain species at a larger scale. Remote imagery, such as satellite, hyperspectral, multispectral, and aerial photography, could detect patterns of defoliation, crown dieback, and tree mortality in forest landscapes. Researchers have begun to explore the application of remote imagery for identifying the distribution or impacts of invasive forest pests, and characterizing their spatial dynamics [38,39,40]. In view of the massive scale of the forested landscapes proposed in the XNA, remote sensing will be a powerful tool for aerial survey of invasive forest pests.

Another useful method for early detection and monitoring is to plant exotic trees as “sentinel trees” in forest landscapes, particularly near botanical gardens, arboreta, and transport linkages [41], which are frequently the first points of contact with exotic pests insects and plant pathogens [42]. Sentinel trees provide an opportunity to detect and eradicate invasive forest pests during the initial stages of establishment, thereby drastically reducing damage, ecological impacts, and long term management costs; however, this strategy needs to include active, regular monitoring on an ongoing basis under the management plan, otherwise these sentinel trees run the risk of invasive species establishment increases.

To raise awareness and encourage involvement from the whole society, it is necessary to further publicize the knowledge of invasive forest pests. For example, a range of local outreach programs and activities can be done to raise awareness of invasive species, such as advocacy of policy and popularization of knowledge, which can be carried out on a regular basis and reiterated at information kiosks at the existing visitor centers, thereby educating potential citizen scientists, whom in turn could utilize the Xiongan Forest APP. In addition, international and interdisciplinary cooperation on data sharing can also greatly contribute to the successful prevention of invasive forest pests in XNA.

The recruitment of citizen scientists will contribute to active monitoring, integrated with the forest protection station-led surveys. In addition, the internet databases can also be widely used by both citizen scientists and the local pest management bureaus to detect and monitor the pests at the early stages of invasion. For example, smartphone applications could be used for day-to-day surveillance of urban tree health, enabling citizen scientists to be on the lookout for potential invaders [43].

### 5.3. Local Eradication

Once an invasive forest pest is discovered during an early detection program or as isolated occurrences in local populations, local eradication should be immediately conducted to prevent its spread. The most common and effective approaches include removal of infested trees, physical control methods, and treatment with pesticides. 

Sanitation, the physical removal of infested trees, eliminates the sources of spread. This method is particularly useful for eradication of wood-boring insects, plant pathogens vectored by wood-boring insects, and other pathogens that inhabit forests [44,45]. The removed plant parts that may harbor live insect or pathogen materials are destroyed by burning, burying, or chipping, so they are not transported out of the infested area. For the insects that oviposit or pupate in a cluster, it is possible to manually remove the egg masses (e.g., moths such as *Lymantria dispar* and *Hyphantria cunea*) [46,47], or pupae masses (e.g., beetle like *Pyrrhalta aenescens*) [48]. These physical control methods will greatly diminish the population of invasive forest pests. Aerial spraying with pesticides is another option for eradication campaigns, and has been used recently in many urban forests [18,49]. Aerial spraying has the advantage of lower costs, good coverage, and effective and quick treatment of a large area. However, concerns about potential toxicity to humans must be properly addressed to mitigate risk to human health. Moreover, this method works well on external feeders like defoliators but is not effective for bark- and wood-boring pests. Thus, we need to further optimize aerial application methods to achieve eradication with minimized costs and environmental impact, yet maintain maximum efficiency.

### 5.4. Long-Term Management

If local eradication is not feasible, then long-term management, including ecosystem and landscape management, biological control, mating disruption, and attract-and-kill will be conducted as the final defense to reduce the extent of the invasion, decrease pest population density, and prevent further expansion within the XNA.

A variety of physical and biological factors in ecosystem processes could affect species invasions. Therefore, integrating invasive species into the conceptual framework for forest ecosystem and landscape management is a good way to eliminate invasion pathways and to minimize impacts where invasive species are established [33]. The “Millennium show forest”, as a new, model approach to forest planning in XNA, provides the opportunity to better integrate invasive species management concerns into regional forest planning and ongoing forest management. Thereby, factors that affect species invasions, e.g., roads, host plants, adjacent land uses, the size of habitat fragments, and the forest edge-to-interior ratio, can all be taken into account during afforestation.

Biological control using predators, pathogens, parasitoids, and herbivores are safe, cost effective and environmentally benign tools for invasive pest management [50]. Many biological control agents for the potential invasive pests in XNA are locally available. For example, *Sclerodermus* sp. and *Dastarcus helophoroides* have been widely used to control *Monochamus alternatus*, the main vector beetle of pinewood nematodes in China. A predator, *Rhizophagus grandis*, can successfully suppress the population of the invasive *Dendroctonus valens* [51]. The genetic modification of some microbial agents such as *Beauveria bassiana* is another promising approach to specifically and efficiently control certain invasive pests.

Semiochemicals such as sex pheromones, aggregation pheromones, kairomones, and allomones play an important role in intra-specific and inter-specific communication of insects, and are also essential components for many insect control techniques [18]. Among these tools, attract-and-kill methods that utilize sex or aggregation pheromones, and/or a kairomone combined with insecticides, are an effective way to reduce insect populations [52,53]. Mating disruption using sex pheromones, is another well-known pest control technique [54]. Push–pull strategies, using repellents to make the protected resource unattractive or unsuitable to the pests (push) and attractants that lure pests and subsequently remove them (pull), can manipulate pest behaviors with a maximized control efficiency and output [55]. For long-term containment of invasive forest pests in XNA, these chemical-based techniques will be considered as insect invaders are detected, eradicated, and/or contained.

In addition to the above strategies, the XNA will have the strategic advantage of having “big data” gathered from the “Millennium show forest” that include detailed information for each tree and overall forest conditions. These data points will be integrated into a database on a cloud-computing network to facilitate real time evaluation of the risks and susceptibility of certain trees, stands, and areas to invasive pests and assist resource managers to make the best decision to strategically manage the urban forest in a way to minimize the potential impacts of invasive species and make them more resilient to invasive species threats.

## 6. Conclusions

The creation of the new city of Xiongan is of great significance. Not only will it relieve the burden of non-capital functions that Beijing currently serves, but it will promote the integration of the Beijing–Tianjin–Hebei region. To meet the concept of the green initiative, a high-quality, large-scale urban forest, termed “Millennium show forest”, is central to the environmental and ecosystem health of the XNA, and will provide a foundation for human health and well-being to the area. However, both native and invasive pest insects and pathogens pose significant threats during the planned urbanization, including the devastating pinewood nematode *B. xylophilus* and red turpentine beetle *D. valens*. To mitigate the impacts of invasive forest pests, we have put forth a set of sequential series of strategies ranging from prevention, early detection and monitoring, and local eradication, to long-term containment, and a decision making system based on a network of “big data”. This new approach to urban forest management, especially integration of the “big data network” with existing early detection and rapid response protocols, will enable managers to minimize the impact of invasive species and provide an ideal model for long-term urban forestry research. Over time, this system will generate data on forest succession and provide a real-time database for prevention, monitoring, and management of pest invasion that has the potential to become a future model system for the world, not only for newly planted forests, but also for existing urban forests.

## Figures and Tables

**Table 1:** Plant species planted in Xiongan New Area.

No.	Families	Genera	Species	Types *
Overstory	Aceraceae	*Acer*	*Acer davidii* Franch.	a
	Aceraceae	*Acer*	*Acer ginnala* Maxim	a
	Aceraceae	*Acer*	*Acer griseum* (Franch.) Pax	a
	Aceraceae	*Acer*	*Acer mono* Maxim	a
	Aceraceae	*Acer*	*Acer negundo* L.	a
	Aceraceae	*Acer*	*Acer platanoides* L.	a
	Aceraceae	*Acer*	*Acer rubrum* L.	a
	Aceraceae	*Acer*	*Acer saccharinum* L.	a
	Aceraceae	*Acer*	*Acer saccharum*	a
	Aceraceae	*Acer*	*Acer saccharinum*	a
	Aceraceae	*Acer*	*Acer triflorum* Komarov	a
	Aceraceae	*Acer*	*Acer truncatum* Bunge	a
	Anacardiaceae	*Pistacia*	*Pistacia chinensis* Bunge	a
	Betulaceae	*Carpinus*	*Carpinus turczaninowii* Hance	a
	Bignoniaceae	*Catalpa*	*Catalpa bungei* C. A. Mey.	a
	Bignoniaceae	*Catalpa*	*Catalpa ovata*	a
	Celastraceae	*Euonymus*	*Euonymus japonicus* Thunb.	a
	Cornaceae	*Swida*	*Swida walteri* (Wanger.) Sojak	a
	Cupressceae	*Juniperus*	*Juniperus rigida* Sieb. and Zucc.	b
	Cupressceae	*Platycladus*	*Platycladus orientalis* (L.) Franco	b
	Cupressceae	*Sabina*	*Sabina chinensis* (L.) Ant.	b
	Ebenaceae	*Diospyros*	*Diospyros kaki* Thunb.	a
	Ebenaceae	*Diospyros*	*Diospyros lotus* L.	a
	Eucommiaceae	*Eucommia*	*Eucommia ulmoides* Oliver	a
	Fagaceae	*Quercus*	*Quercus acutissima* Carruth.	a
	Fagaceae	*Quercus*	*Quercus aliena* Bl.	a
	Fagaceae	*Quercus*	*Quercus dentata* Thunb.	a
	Fagaceae	*Castanea*	*Castanea mollissima* Bl.	a
	Fagaceae	*Quercus*	*Quercus mongolica* Fisch. ex Ledeb	a
	Fagaceae	*Quercus*	*Quercus variabilis* Bl.	a
	Fagaceae	*Quercus*	*Quercus wutaishansea* Mary	a
	Flacourtiaceae	*Idesia*	*Idesia polycarpa* Maxim.	a
	Ginkgoaceae	*Ginkgo*	*Ginkgo biloba* L.	a
	Hippocastanaceae	*Aesculus*	*Aesculus chinensis*	a
	Juglandaceae	*Juglans*	*Juglans mandshurica*	a
	Juglandaceae	*Juglans*	*Juglans regia* L.	a
	Juglandaceae	*Pterocarya*	*Pterocarya stenoptera*	a
	Leguminosae	*Albizia*	*Albizia julibrissin* Durazz.	a
	Leguminosae	*Gleditsia*	*Gleditsia sinensis* Lam.	a
	Leguminosae	*Robinia*	*Robinia pseudoacacia* L.	a
	Leguminosae	*Sophora*	*Sophora japonica* Linn.	a
	Leguminosae	*Sophora*	*Robinia pseudoacacia* cv. Idaho	a
	Magnoliaceae	*Liriodendron*	*Liriodendron chinense* (Hemsl.) Sarg.	a
	Magnoliaceae	*Liriodendron*	*Liriodendron × sinoamericanum* P.C. Yieh ex C.B. Shang and Zhang R. Wang	a
	Magnoliaceae	*Magnolia*	*Michelia alba*	a
	Magnoliaceae	*Magnolia*	*Magnolia biondii* Pamp.	a
	Magnoliaceae	*Magnolia*	*Magnolia denudata* Desr.	a
	Magnoliaceae	*Magnolia*	*Magnolia grandiflora* L	a
	Magnoliaceae	*Magnolia*	*Magnolia soulangeana* Soul.-Bod.	a
	Moraceae	*Morus*	*Morus alba* L.	a
	Meliaceae	*Melia*	*Melia azedarach* L.	a
	Meliaceae	*Toona*	*Toona sinensis*	a
	Oleaceae	*Coptosapelta*	*Chionanthus retusus*	a
	Oleaceae	*Fraxinus*	*Fraxinus americana* “Autumn Purple”	a
	Oleaceae	*Fraxinus*	*Fraxinus bungeana* DC	a
	Oleaceae	*Fraxinus*	*Fraxinus chinensis* Roxb	a
	Oleaceae	*Fraxinus*	*Fraxinus mandshurica* Rupr.	a
	Oleaceae	*Fraxinus*	*Fraxinus pennsylvanica* Marsh.	a
	Oleaceae	*Fraxinus*	*Fraxinus velutina* Torr	a
	Pinaceae	*Cedrus*	*Cedrus deodara* (Roxb.) G. Don	b
	Pinaceae	*Picea*	*Picea asperata* Mast.	b
	Pinaceae	*Picea*	*Picea meyeri* Rehd. and Wils.	b
	Pinaceae	*Picea*	*Picea wilsonii* Mast.	b
	Pinaceae	*Pinus*	*Pinus armandii* Franch.	b
	Pinaceae	*Pinus*	*Pinus bungeana* Zucc. ex Endl	b
	Pinaceae	*Pinus*	*Pinus ponderosa* Dougl. ex Laws.	b
	Pinaceae	*Pinus*	*Pinus strobus* L.	b
	Pinaceae	*Pinus*	*Pinus sylvestris* L var. *mongolica* Litv	b
	Pinaceae	*Pinus*	*Pinus tabuliformis* Carr.	b
	Pinaceae	*Pinus*	*Pinus thunbergii* Parl.	b
	Platanaceae	*Platanus*	*Platanus orientalis* Linn.	a
	Rhamnaceae	*Hovenia*	*Hovenia acerba* Thunb.	a
	Rosaceae	*Amygdalus*	*Amygdalus persica* L.	a
	Rosaceae	*Amygdalus*	*Amygdalus persica* L.var. persica f. *atropurpurea* Schneid.	a
	Rosaceae	*Armeniaca*	*Armeniaca sibirica* (L.) Lam.	a
	Rosaceae	*Armeniaca*	*Armeniaca vulgaris* Lam.	a
	Rosaceae	*Cerasus*	*Cerasus pseudocerasus* (Lindl.) G. Don	a
	Rosaceae	*Cerasus*	*Cerasus serrulata*	a
	Rosaceae	*Cerasus*	*Cerasus serrulata* (Lindl.) G. Don ex London var. *lannesiana* (Carri.) Makino	a
	Rosaceae	*Cerasus*	*Cerasus subhirtella* (Miq.) Sok	a
	Rosaceae	*Cerasus*	*Cerasus tomentosa* (Thunb.) Wall.	a
	Rosaceae	*Cerasus*	*Cerasus yedoensis* (Matsum.) Yu and Li	a
	Rosaceae	*Malus*	*Malus halliana* Koehne	a
	Rosaceae	*Malus*	*Malus micromalus* cv. “American”	a
	Rosaceae	*Malus*	*Malus micromalus* cv. “Ruby”	a
	Rosaceae	*Malus*	*Malus* “Radiant”	a
	Rosaceae	*Malus*	*Malus robusta* Rehd	a
	Rosaceae	*Padus*	*Prunus wilsonii*	a
	Rosaceae	*Prunus*	*Prunus cerasifera* Ehrhar f. *atropurpurea* (Jacq.) Rehd.	a
	Rosaceae	*Prunus*	*Prunus sargentii* Rehd	a
	Rosaceae	*Pyrus*	*Pyrus betulifolia* Bunge	a
	Rosaceae	*Sorbus*	*Sorbus alnifolia* (Sieb. and Zucc.) K. Koch	a
	Rutaceae	*Phellodendron*	*Phellodendron amurense* Rupr.	a
	Salicaceae	*Populus*	*Populus alba* var. *pyramidalis* Bge.	a
	Salicaceae	*Populus*	*Populus* × *beijingensis* W. Y. Hsu	a
	Salicaceae	*Populus*	*Populus hopeiensis* Hu and Chow	a
	Salicaceae	*Populus*	*Populus tomentosa* Carrière	a
	Salicaceae	*Salix*	*Salix babylonica*	a
	Salicaceae	*Salix*	*Salix matsudana* var. *matsudana* f. *umbraculifera* Rehd.	a
	Salicaceae	*Salix*	*Salix matsudana* Koidz	a
	Salicaceae	*Salix*	*Salix matsudana* var. *matsudanan* f. *pendula* Schneid	a
	Salicaceae	*Salix*	*Salix capitata* Y. L. Chou and Skv	a
	Sapindaceae	*Koelreuteria*	*Koelreuteria paniculata* Laxm.	a
	Scrophulariaceae	*Paulownia*	*Paulownia fortunei*	a
	Scrophulariaceae	*Paulownia*	*Paulownia tomentosa* (Thunb.) Steud.	a
	Simaroubaceae	*Ailanthus*	*Ailanthus altissima* “Qiantou”	a
	Simaroubaceae	*Ailanthus*	*Ailanthus altissima* (Mill.) Swingle	a
	Sterculiaceae	*Firmiana*	*Firmiana platanifolia* (L. f.) Marsili	a
	Tamaricaceae	*Tamarix*	*Tamarix chinensis* Lour.	a
	Taxodiaceae	*Metasequoia*	*Metasequoia glyptostroboides* Hu and W. C. Cheng	a
	Tiliaceae	*Tilia*	*Tilia amurensis* Rupr	a
	Tiliaceae	*Tilia*	*Tilia mandshurica* Rup and Maxim.	a
	Tiliaceae	*Tilia*	*Tilia tomentosa* Moench	a
	Tiliaceae	*Tilia*	*Tilia tuan* Szyszyl.	a
	Ulmaceae	*Celtis*	*Celtis bungeana* Bl.	a
	Ulmaceae	*Celtis*	*Celtis koraiensis* Nakai	a
	Ulmaceae	*Celtis*	*Celtis sinensis* Pers.	a
	Ulmaceae	*Pteroceltis*	*Pteroceltis tatarinowii* Maxim.	a
	Ulmaceae	*Ulmus*	*Ulmus davidiana* Planch.	a
	Ulmaceae	*Ulmus*	*Ulmus lamellosa* T. Wang and S. L. Chang ex L. K. Fu	a
	Ulmaceae	*Ulmus*	*Ulmus macrocarpa* Hance	a
	Ulmaceae	*Ulmus*	*Ulmus parvifolia* Jacq	a
	Ulmaceae	*Ulmus*	*Ulmus pumila* L.	a
	Ulmaceae	*Ulmus*	*Ulmus pumila* cv.jinye	a
	Ulmaceae	*Zelkova*	*Zelkova serrata* (Thunb.) Makino	a
Mid-canopy	Anacardiaceae	*Cotinus*	*Cotinus coggygria* Scop	a/c
	Celastraceae	*Euonymus*	*Euonymus maackii* Rupr.	a/c
	Cornaceae	*Macrocarpium*	*Cornus officinalis* Sieb. and Zucc.	a/c
	Moraceae	*Cudrania*	*Cudrania tricuspidata* (Carr.) Bur. ex Lavallee	a/c
	Oleaceae	*Syringa*	*Syringa oblata* Lindl.	a/c
	Oleaceae	*Syringa*	*Syringa pekinensis* Rupr.	a/c
	Oleaceae	*Syringa*	*Syringa reticulata* (Bl.) Hara var. *mandshurica* (Maxim.) Hara (*S. amurensis* Rupr.)	a/c
	Rhamnaceae	*Rhamnus*	*Rhamnus davurica* Pall.	a/c
	Rosaceae	*Malus*	*Malus baccata* (L.) Borkh	a/c
	Sapindaceae	*Xanthoceras*	*Xanthoceras sorbifolia* Bunge	a/c
Understory	Caprifoliaceae	*Abelia*	*Abelia biflora* Turcz.	c
	Caprifoliaceae	*Kolkwitzia*	*Kolkwitzia amabilis* Graebn.	c
	Caprifoliaceae	*Lonicera*	*Lonicera maackii* (Rupr.) Maxim.	c
	Caprifoliaceae	*S* *ambuceae*	*Sambucus williamsii* Hance	c
	Caprifoliaceae	*Viburnum*	*Viburnum opulus* Linn. var. *calvescens* (Rehd.) Hara	c
	Caprifoliaceae	*Weigela*	*Weigela florida* (Bunge) A. DC.	c
	Caprifoliaceae	*Weigela*	*Weigela florida* cv. Red Prince	c
	Celastraceae	*Euonymus*	*Euonymus bungeanus* Maxim.	c
	Celastraceae	*Euonymus*	*Euonymus kiautschovicus*	c
	Cupressceae	*Sabina*	*Sabina vulgaris* Ant.	c
	Leguminosae	*Amorpha*	*Amorpha fruticosa* Linn.	c
	Leguminosae	*Cercis*	*Cercis chinensis* Bunge	c
	Leguminosae	*Cercis*	*Cercis glabra* Pampan.	c
	Leguminosae	*Lespedeza*	*Lespedeza bicolor* Turcz	c
	Lythraceae	*Lagerstroemia*	*Lagerstroemia indica* L.	c
	Malvaceae	*Hibiscus*	*Hibiscus syriacus* Linn.	c
	Oleaceae	*Forsythia*	*Forsythia suspensa* (Thunb.) Vahl	c
	Oleaceae	*Jasminum*	*Jasminum nudiflorum* Lindl.	c
	Oleaceae	*Ligustrum*	*Ligustrum compactum* (Wall. ex G. Don) Hook.f. and Thomson ex Decne.	c
	Oleaceae	*Ligustrum*	*Ligustrum quihoui* Carr.	c
	Oleaceae	*Syringa*	*Syringa persica* var. *laciniata* West	c
	Oleaceae	*Syringa*	*Syringa villosa* Vahl	c
	Rhamnaceae	*Rhamnus*	*Rhamnus arguta* Maxim	c
	Rhamnaceae	*Rhamnus*	*Rhamnus globosa* Bunge	c
	Rosaceae	*Amygdalus*	*Amygdalus triloba*	c
	Rosaceae	*Chaenomeles*	*Chaenomeles*	c
	Rosaceae	*Chaenomeles*	*Chaenomeles cathayensis* Schneid.	c
	Rosaceae	*Chaenomeles*	*Chaenomeles speciosa* (Sweet) Nakai	c
	Rosaceae	*Cotoneaster*	*Cotoneaster horizontalis* Decne	c
	Rosaceae	*Cotoneaster*	*Cotoneaster amoene* Wils.	c
	Rosaceae	*Kerria*	*Kerria japonica* (L.) DC	c
	Rosaceae	*Physocarpus*	*Physocarpus amurensis* (Maxim.) Maxim.	c
	Rosaceae	*Rosa*	*Rosa xanthina* Lindl.	c
	Rosaceae	*Sorbaria*	*Sorbaria sorbifolia* (L.) A. Br.	c
	Rosaceae	*Sorbaria*	*Sorbaria kirilowii* (Regel) Maxim.	c
	Saxifragaceae	*Deutzia*	*Deutzia parviflora* Bge.	c
	Saxifragaceae	*Deutzia*	*Deutzia scabra* Thunb	c
	Saxifragaceae	*Hydrangea*	*Hydrangea macrophylla* (Thunb.) Ser.	c
	Saxifragaceae	*Philadelphus*	*Philadelphus pekinensis* Rupr.	c

* Types include a. deciduous tree, b. evergreen tree, and c. shrub.

**Table 2:** Existing pests, including insects and diseases, in Xiongan New Area.

Pest Type	Tissues Attacked/Damaged	Species	Host Plants	Extent of Damages *
**Insects**	Trunk and branch	*Semanotus bifasciatus*	*Sabina chinensis*	35.40%
	Trunk	*Holcocerus insularis*	*Fraxinus* sp.	5.10%
	Trunk and branch	*Anoplophora glabripennis*	*Acer ginnala*, *Populus beijingensis*, *Acer palmatum*, and *Salix* sp.	4.50%
	Leaf	*Semiothisa cinerearia*	*Sophora japonica*	13.50%
	One-year branch	*Omphisa plagialis*	*Catalpa bungei*	11.00%
	Leaf	Aphidoidea	*Fraxinus* sp., *Sophora japonica*, *Euonymus maackii*, *Pyrus* sp. and *Cerasus* sp.	13.20%
	Leaf	*Thysanogyna limbata*	*Firmiana platanifolia*	23.40%
	Leaf	*Cnidocampa flavescens*	*Pyrus* sp. and *Acer truncatum*	12.00%
	Leaf	*Hyphantria cunea*	*Morus* sp., *Salix* sp., *Fraxinus* sp. and *Pyrus* sp.	23.40%
	Spring tip	*Dioryctria rubella*	*Pinus tabuliformis*	20.70%
	Leaf	*Pyrrhalta aenescens*	*Ulmus* sp.	7.10%
	Leaf	*Plagiodera versicolora*	*Populus* sp. and *Salix* sp.	5.90%
	New tip	*Aphrophora costalis*	*Salix* sp.	8.90%
	Leaf	*Panonychus ulmi*	*Pyrus* sp.	20.50%
	Leaf	*Aculops niphocladae*	*Salix* sp.	3.90%
	Leaf	*Malacosoma neustria testacea*	*Populus* sp.	1.20%
	Leaf	*Clostera anachoreta*	*Populus* sp.	1.50%
	New tip	*Spilonota lechriaspis*	*Pyrus* sp. and *Amygdalus davidiana*	4.50%
	Branch	*Cryptotympana atrata*	*Fraxinus* sp. and *Acer truncatum*	2.30%
**Diseases**	Leaf	Juniper rust disease	*Sabina chinensis* and *Pyrus* sp.	6.50%
	Trunk and branch	Rot disease	*Pyrus* sp., *Sophora japonica*, *Populus* sp., and *Salix* sp.	3.40%
	Trunk and branch	ulcer disease	*Populus* sp., *Sophora japonica*, and *Juglans* sp.	3.50%
	Root	Root crown gall	*Cerasus* sp.	10.10%
	Whole plant	Fusarium wilt	*Cotinus coggygria*, *Acer truncatum*, and *Gleditsia sinensis*	2.80%
	Leaf	powdery mildew	*Populus* sp. and *Salix* sp.	14.00%
	Leaf	Leaf spot disease	*Pyrus* sp. and *Syringa* sp.	10.50%
	Leaf	Leaf black spot disease	*Pyrus* sp., *Syringa* sp., and *Malus* sp.	12.50%
	Leaf	Leaf rust disease	*Populus* sp. and *Salix* sp.	13.90%
	Leaf	Pine needle cast	*Pinus tabuliformis*	15.80%
	Leaf	Pine red blight	*Pinus tabuliformis*	12.10%
	Leaf	Leaf blight	*Ginkgo biloba*	15.20%
	Leaf	black spot disease	*Rosa chinensis*, *Rosa xanthina*	5.80%
	Trunk and branch	Gummosis disease	*Prunus* sp. and *Prunus ceraifera*	3.10%
	Leaf	Leaf spot disease	*Paeonia suffruticosa, Paeonia lactiflora*	16.50%
	Leaf	anthracnose disease	*Euonymus japonzcus*	10.10%
	Leaf	Bacterial shot hole disease	*Prunus* sp.	3.50%
	Leaf	Iron-deficiency yellow disease	*Malus spectabilis*	15.90%

* The surveys of damage extent were conducted during 2018–2019. For each pest, we set up three standard sample plots, and 100 trees in each plot were randomly selected and individually investigated. The extent of damage was then calculated in percentage. Overall, this assemblage of pests and pathogens will bring threats to different plant parts of many different tree species. They form a local pest repository and could be sources of pest resurgence following control measures, and have the potential to become invasive once carried or transmitted to the new millennium forest surrounding the XNA.
